# Partial Deficiency of Sphingosine-1-Phosphate Lyase Confers Protection in Experimental Autoimmune Encephalomyelitis

**DOI:** 10.1371/journal.pone.0059630

**Published:** 2013-03-27

**Authors:** Andreas Billich, Thomas Baumruker, Christian Beerli, Marc Bigaud, Christian Bruns, Thomas Calzascia, Andrea Isken, Bernd Kinzel, Erika Loetscher, Barbara Metzler, Matthias Mueller, Barbara Nuesslein-Hildesheim, Bernadette Kleylein-Sohn

**Affiliations:** Novartis Institutes for BioMedical Research, Basel, Switzerland; University of Muenster, Germany

## Abstract

**Background:**

Sphingosine-1-phosphate (S1P) regulates the egress of T cells from lymphoid organs; levels of S1P in the tissues are controlled by S1P lyase (Sgpl1). Hence, Sgpl1 offers a target to block T cell-dependent inflammatory processes. However, the involvement of Sgpl1 in models of disease has not been fully elucidated yet, since Sgpl1 KO mice have a short life-span.

**Methodology:**

We generated inducible Sgpl1 KO mice featuring partial reduction of Sgpl1 activity and analyzed them with respect to sphingolipid levels, T-cell distribution, and response in models of inflammation.

**Principal Findings:**

The partially Sgpl1 deficient mice are viable but feature profound reduction of peripheral T cells, similar to the constitutive KO mice. While thymic T cell development in these mice appears normal, mature T cells are retained in thymus and lymph nodes, leading to reduced T cell numbers in spleen and blood, with a skewing towards increased proportions of memory T cells and T regulatory cells. The therapeutic relevance of Sgpl1 is demonstrated by the fact that the inducible KO mice are protected in experimental autoimmune encephalomyelitis (EAE). T cell immigration into the CNS was found to be profoundly reduced. Since S1P levels in the brain of the animals are unchanged, we conclude that protection in EAE is due to the peripheral effect on T cells, leading to reduced CNS immigration, rather than on local effects in the CNS.

**Significance:**

The data suggest Sgpl1 as a novel therapeutic target for the treatment of multiple sclerosis.

## Introduction

Sphingosine-1-phosphate (S1P) is a pluripotent lipid signaling molecule with important functions in health and disease across a broad range of organ systems [1]–[4]. S1P has been well characterized as an agonist of five G-protein coupled receptors, named S1P_1_ to S1P_5_ [5], [6]. Among these receptors, S1P_1_ is of particular interest as a target in immunomodulation; the drug fingolimod (FTY720, Gilenya™), licensed for the treatment of relapsing multiple sclerosis, acts in its phosphorylated form as S1P_1_ modulator and thus regulates the migration of selected lymphocyte subsets into the central nervous system [7]. More recently, direct intracellular targets of S1P have been characterized that may offer additional points for pharmacological intervention [8], [9]. As opposed to interfering with the molecular targets of S1P, modulation of its concentration constitutes an alternative approach to capture the therapeutic benefit of inhibiting or enhancing the functions of S1P. This appears achievable in at least three different ways: (i) by using anti-S1P antibodies to reduce extracellular S1P [10]; (ii) by inhibiting or enhancing the activity of intracellular sphingosine kinases which produce S1P [11], [12]; (iii) by blocking S1P-degrading enzymes, namely the S1P phosphatases or S1P lyase [13]. Drug candidates from all three approaches, namely an S1P antibody [10], sphingosine kinase inhibitors [14], [15], and a lyase inhibitor [16], [17], are currently under evaluation in clinical trials.

S1P lyase (Sgpl1), a microsomal enzyme ubiquitously expressed in mammalian tissues, is engaged in the irreversible degradation of S1P to 2-hexadecenal and phosphoethanolamine [13], [18]. Thus, this enzyme is considered to be a major control point to regulate S1P concentrations in cells. Indeed, constitutive knock-out of Sgpl1 in mice leads to a pronounced increase of S1P levels in tissues and serum [19]; new-born Sgpl1 KO mice do not thrive, feature major derailment of lipid metabolism and innate immune functions, and die early in life [19]–[22]. However, partial inhibition of Sgpl1, which may lead to less pronounced and more benign increases of S1P levels, has been proposed as a therapeutic modality, in particular in autoimmune disease [16], [19], [23]–[25]. As originally observed by J. Cyster and co-workers [26], Sgpl1 is required to maintain an S1P gradient between tissues (low S1P) on the one hand and efferent lymph and blood (high S1P) on the other, which appears to be required for the T cell egress from the lymphoid organs. Indeed, reduced numbers of T cells in the circulation are a consistent observation in mice completely or partially deficient in Sgpl1 activity [19], or in rodents treated with Sgpl1 inhibitors, such as 2-acetyl-4(5)-tetrahydroxybutyl imidazole (THI) or LX-2931 ( = LX3305) [16], [27]. The latter compound was also efficacious in reducing peripheral T cell numbers in healthy subjects in the course of a clinical phase I study [16]; a phase II study in RA failed to meet its primary endpoint, apparently due to subtherapeutic dosing [17].

To date, the therapeutic potential of Sgpl1 inhibitors has not been fully explored. Therefore, we sought to establish a genetic model of partial Sgpl1 deficiency without the limitations of constitutive KO mice [19], [20]. Here we describe a mouse strain in which Sgpl1 gene deletion is inducible in the adult animal, leading to partial reduction of enzyme activity. Importantly, these mice feature pronounced reduction of peripheral T lymphocyte counts and are fully protected in a model of experimental autoimmune encephalomyelitis. This indicates that inhibiting Sgpl1 may represent a new treatment strategy for autoimmune diseases including multiple sclerosis.

## Results

### Partially Sgpl1-deficient Mice Survive after Induced Knock-out

We established mouse strains with either constitutive or inducible KO of Sgpl1. First, a mouse line was prepared in which exon 8 of the Sgpl1-encoding gene is flanked by loxP elements (Fig. S1). Crossing of the floxed Sgpl1 mice with a Cre deleter line yielded the constitutive KO mice. To generate inducible Sgpl1 KO mice, the floxed Sgpl1 mice were crossed with a B6.C actb-CreERT2 knock-in mouse line [28]; breeding yielded Sgpl1^Flox/Flox^ Cre^+/−^ ( = inducible KO) and Sgpl1^Flox/Flox^ Cre^−/−^ ( = control) littermates, which were used for experimentation.

**Figure 1:**
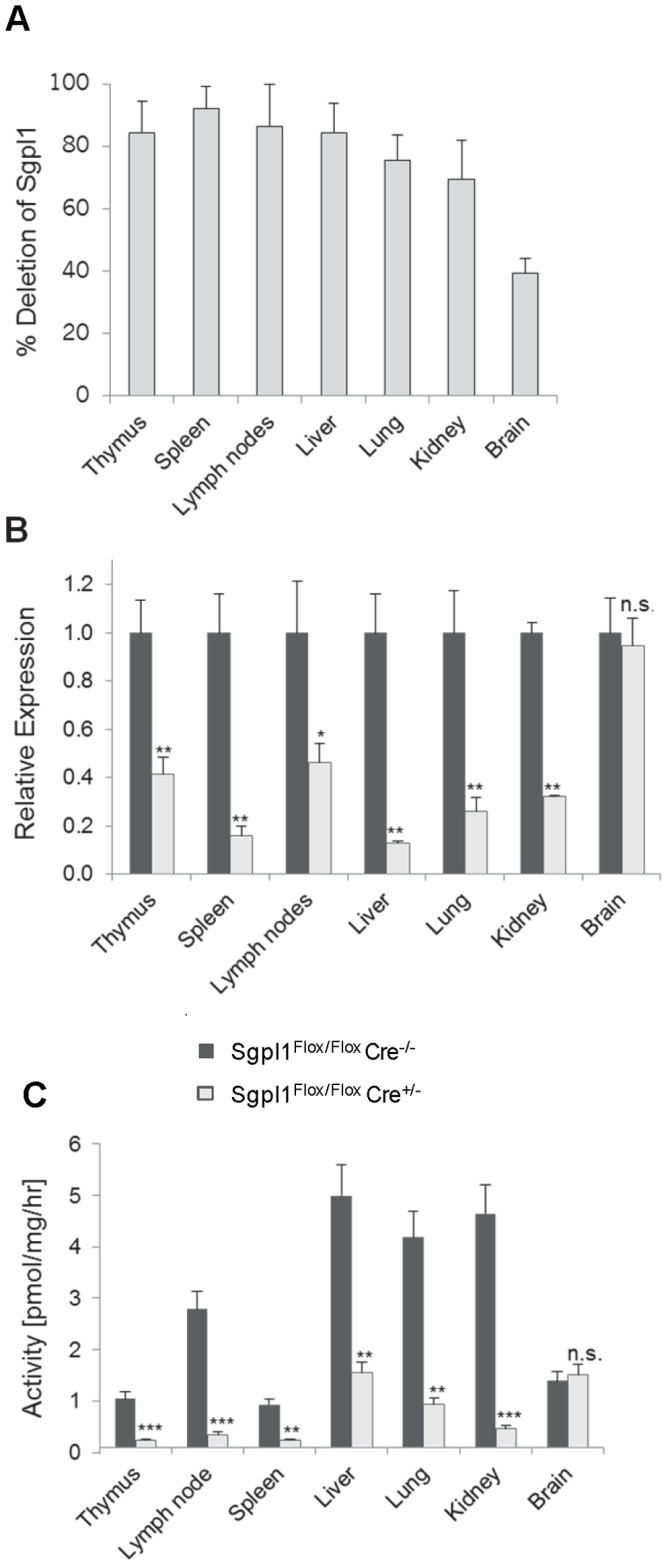
Sgpl1 expression in induced Sgpl1^Flox/Flox^ Cre^+/−^ mice. Two weeks after tamoxifen treatment, tissues from both Cre-positive and negative Sgpl1^Flox/Flox^ mice (4 males and females, each) were analysed. *A,* Genomic DNA was isolated and analysed by RT-PCR probing for precence of exon 8. The percentage of gene deletion as compared to Sgpl1^Flox/Flox^ Cre^−/−^ controls is given. *B*, mRNA was isolated and analysed by RT-PCR; expression levels in both the Sgpl1^Flox/Flox^ Cre^−/−^ controls (filled bars) (set to 1 for each organ) and in the induced Sgpl1^Flox/Flox^ Cre^+/−^ mice (open bars) are given. *C,* Sgpl1 activity is tissue homogenates was measured using 15-NBD-S1P as substrate. Activity is reported as product formed per mg of wet tissue per hour. Sgpl1^Flox/Flox^ Cre^−/−^ controls: filled bars; induced Sgpl1^Flox/Flox^ Cre^+/−^: open bars.

Sgpl1^Flox/Flox^Cre^+/−^ mice were treated with tamoxifen for 5 days to induce Sgpl1 knock-out; two weeks later, deletion of exon 8 was observed with a frequency of 70–90% in the genomic DNA of various tissues, with the notable exception of brain (∼40%) (Fig. 1A). Consequently, Sgpl1 mRNA and enzyme activity were reduced by 60 to 90% as compared to control mice, with the exception of brain where no decrease occurred (Fig. 1B,C). Thus, while Sgpl1 gene expression was markedly downregulated, significant residual enzyme activity was still present in these mice. As a consequence, the partially Sgpl1-deficient inducible KO mice showed normal weight gain and survival during a 6 month observation period, in contrast to fully Sgpl1-deficient constitutive KO mice which after birth did not gain weight and died within 3 to 4 weeks. Thus, the inducible KO mice are much better suited for experimentation than the constitutive KO mice.

### Partially Sgpl1-deficient Mice show Less Increase in Sphingolipids, None in Brain

**Figure 2:**
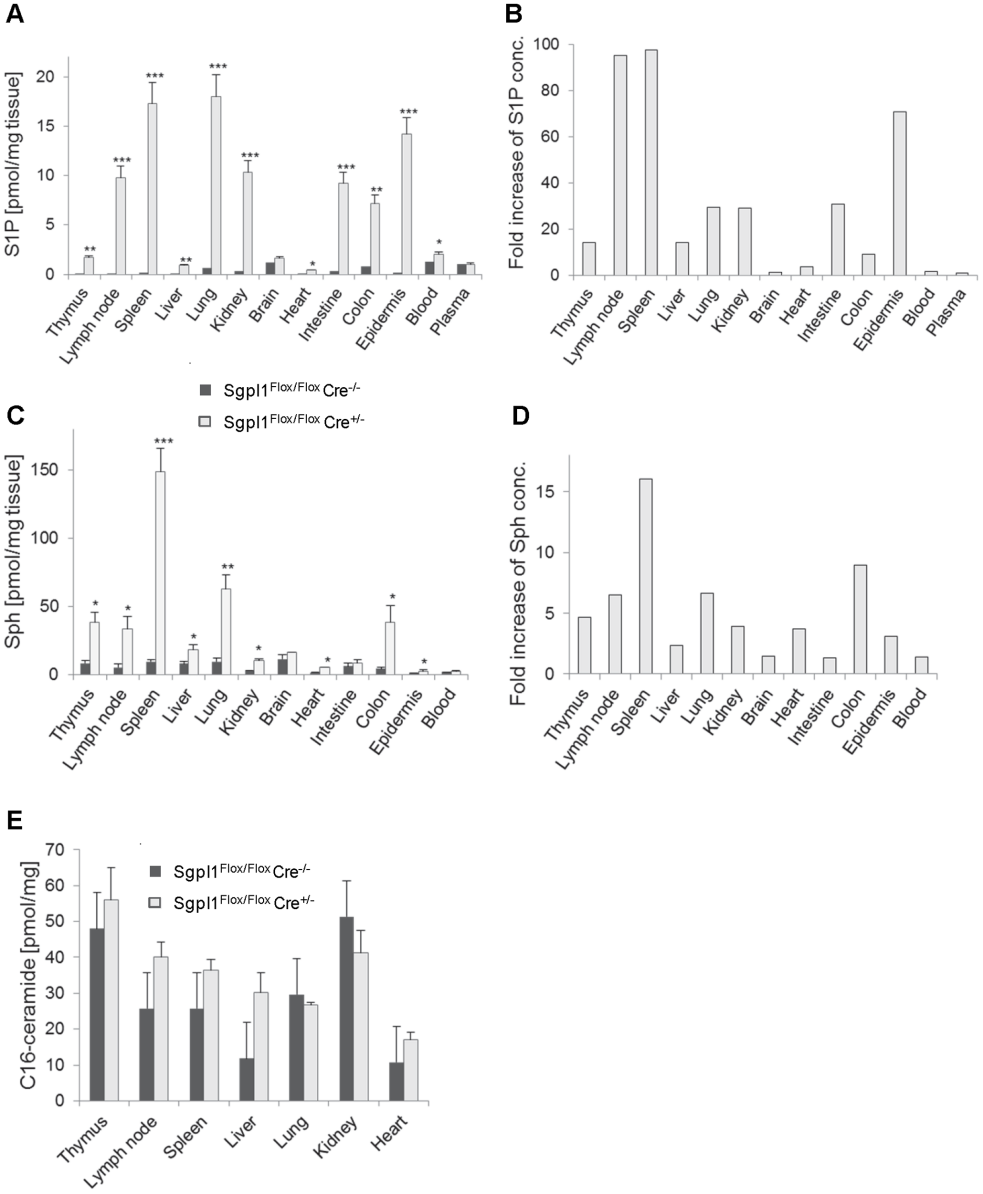
Sphingolipid concentration in selected tissues of inducible Sgpl1-deficient mice. Two weeks after tamoxifen induction, tissues of Sgpl1^Flox/Flox^ Cre^+/−^ mice (open bars) and of Sgpl1^Flox/Flox^ Cre^−/−^ controls (filled bars) were obtained (n = 5/group). Tissues were extracted and sphingolipids were quantified by LC/MS. *A, B,* S1P; *C, D*, Sph; *E,* C16-ceramide. *A, C,* and *E* show absolute concentrations per weight of tissue; *B and D* show fold increase in inducible KO mice.

We compared the effect of complete and partial Sgpl1-deficiency on the Sgpl1 substrate S1P and its metabolic precursors sphingosine (Sph) and ceramide. While new-born constitutive Sgpl1-deficient mice showed almost normal S1P concentrations (data not shown), S1P determined two weeks after birth was increased by factors ranging from 120-fold (lung) to 4700-fold (thymus) (Fig. S2A,B). In inducible Sgpl1-deficient mice, analyzed two weeks after induction of gene deletion, S1P in the tissues was elevated to a lesser degree, ranging from 4-fold (heart) to 100-fold (lymph nodes and spleen) (Fig. 2A,B). In both mouse strains, there was no statistically significant increase of S1P in the brain and spinal cord. Concentrations of S1P in the blood of the inducible Sgpl1-deficient mice showed a moderate elevation (factor of 1.6), while plasma concentrations were not increased at all (Fig. 2A). S1P concentrations in inducible Sgpl1-deficient mice were similar at early time points and 6 months after induction (data not shown). Sph was increased more strongly in constitutive Sgpl1-deficient mice (up to 200-fold; Fig. S2C,D) than in inducible Sgpl1-deficient mice (up to 16-fold; Fig. 2C,D). Furthermore, while C16-ceramide was elevated in the constitutive Sgpl1-deficient mice up to 9-fold in selected tissues (Fig. S2E), there was only a trend towards C16-ceramide increase in the inducible Sgpl1-deficient mice (Fig. 2E). In summary, inducible Sgpl1-deficient mice feature less pronounced increase of sphingolipid metabolites as compared to the constitutive KO mice.

### Reduced Blood T Cell Numbers in Inducible Sgpl1-deficient Mice

**Figure 3:**
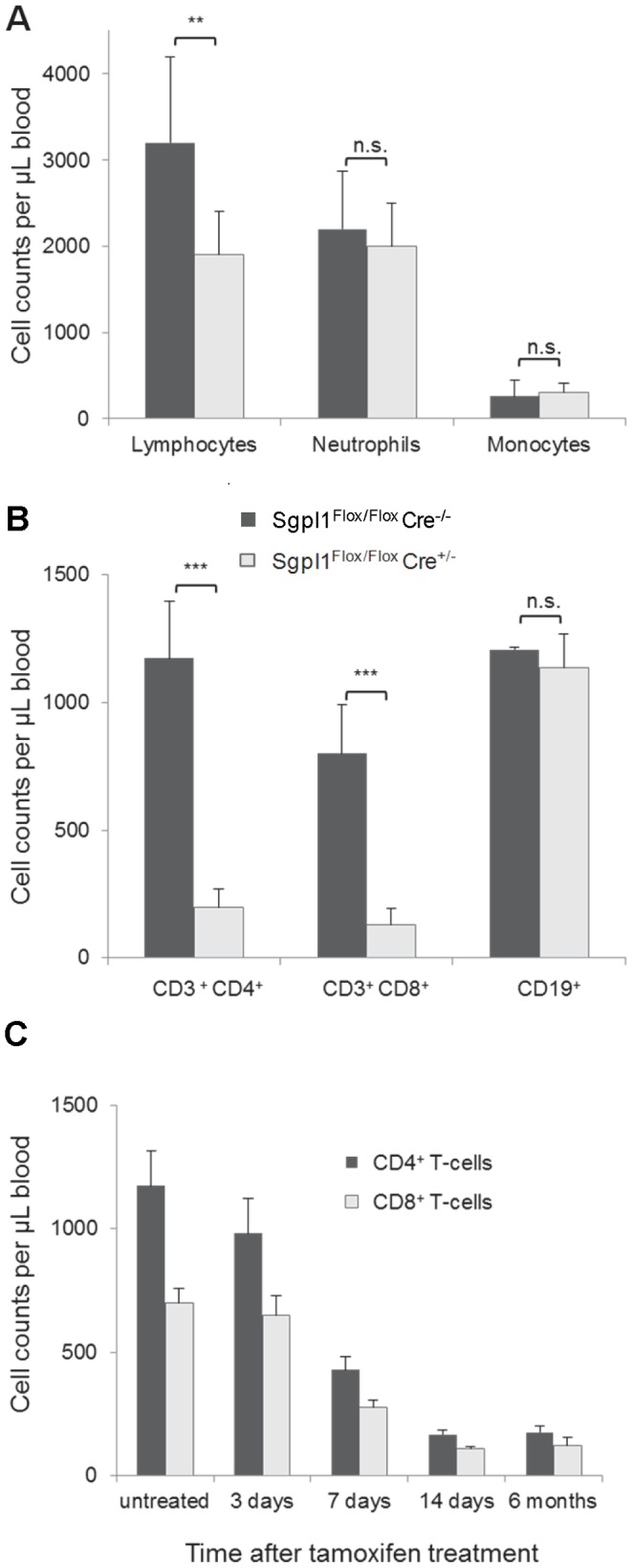
Blood cell numbers in inducible Sgpl1-deficient mice. Mice were treated on 5 consecutive days with 40 mg/kg tamoxifen p.o. *A, B,* Two weeks after tamoxifen treatment of Sgpl1^Flox/Flox^ Cre^+/−^ mice (open bars) and of Sgpl1^Flox/Flox^ Cre^−/−^ controls (closed bars) (n = 5/group), differential blood cell counts were determined (*A*) and FACS analysis of lymphocyte subsets was performed (*B*). *C*, Time course of blood T cell number reduction in induced Sgpl1^Flox/Flox^ Cre^+/−^ mice. (n = 5/group). Blood samples were analysed at the indicated time points by FACS with staining for CD3/CD4 (closed bars) and CD3/CD8-double positive T cells (open bars).

Numbers of neutrophils, monocytes, platelets, and erythrocytes in the blood of inducible Sgpl1-deficient mice were not significantly different from control mice (Fig. 3A and data not shown). In contrast, blood lymphocytes were reduced by 40% (Fig. 3A). This was due to strongly reduced CD4- and CD8-positive T cells by approximately 85%, while CD19-positive B cells were unaffected (Fig. 3B). The decrease of T cell numbers was similar in constitutive KO mice. Tamoxifen treatment of Sgpl1^Flox/Flox^Cre^−/−^ controls and of unfloxed Cre^+/−^ and Cre^−/−^ mice did not affect T cell numbers, indicating that the effect was strictly dependent on recombination of Sgpl1. As for the time course of reduction of T-cell numbers in Sgpl1^Flox/Flox^Cre^+/−^ mice, five daily doses of tamoxifen (40 mg/kg p.o.) yielded maximal decrease of both CD4- and CD8-positive T cells two weeks later, which lasted for at least 6 months (Fig. 3C). T cells were less strongly reduced after fewer doses of tamoxifen or a shortened waiting period (data not shown). Collectively, these data indicate a selective effect of partial Sgpl1 deficiency on peripheral T cell numbers.

### Normal Thymic T Cell Development in Partially Sgpl1-deficient Mice, but Retention in Thymus and LN

**Figure 4:**
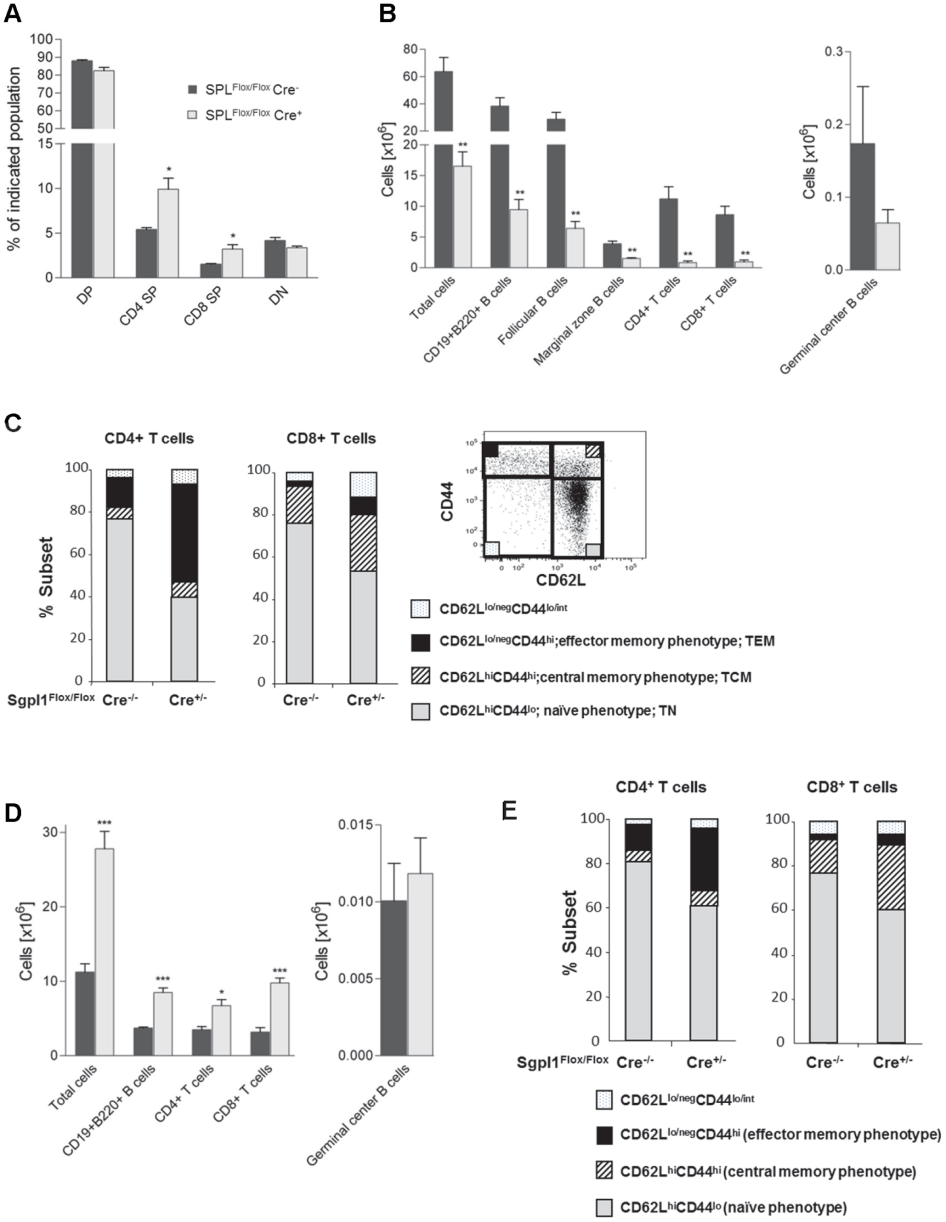
Normal T cell development, reduced splenic cellularity, and increased LN cell number in inducible Sgpl1-deficient mice. B and T cell subpopulations in tamoxifen-treated Sgpl1^Flox/Flox^Cre^+/−^ (open bars) and Sgpl1^Flox/Flox^ Cre^−/−^ mice (closed bars) (n = 4/group), were enumerated based on total live cell counts and cell proportions as established by flow cytometry. *A*, Thymus; *B, C*, spleen; *D, E* lymph nodes. In *C* and *E*, CD8 and CD4 T cells were analysed for co-expression of CD44 and CD62L to define populations of naive and memory T cells; the insert in *C* provides a gating example for naïve/memory type T cells.

In view of the strongly diminished T cell numbers in the blood of the inducible Sgpl1-deficient mice we next studied thymocyte subsets and the composition of lymphocytes in spleen and LN. At 8 weeks following tamoxifen treatment, thymocytes were enriched in mature single positive (SP) CD4 and CD8 cells (Fig. 4A), although the absolute numbers of most thymocyte subsets, including double-negative (DN) cells were not significantly affected (Fig. S3A).

In contrast, in spleen and LN partial Sgpl1 deficiency affected both the size and composition of several lymphocyte subsets. In particular, the splenic cellularity strongly declined, including major reductions in the overall numbers of both CD4- and CD8-positive T cells and subpopulations of B cells (Fig. 4B). While the proportional representation of B cell subsets was not affected by Sgpl1 deficiency (Fig. S3B), there was a sharp decline in the total numbers of follicular and marginal zone B cells, albeit no statistically significant loss of the physiologically small numbers of germinal center B cells was seen (Fig. 4B). The profoundly reduced pools of splenic CD4- and CD8-positive T cells were strongly skewed towards cells of a memory phenotype, in particular CD4 T effector memory and CD8 central memory cells (Fig. 4C).

In contrast to spleen, LN cell numbers were significantly increased in Sgpl1^Flox/Flox^Cre^+/−^ mice compared to control mice. Larger LN cellularity was associated with a significant increase in CD4 and CD8 positive T cells, as well as total B cell numbers (with the exception of germinal center B cells) (Fig. 4D). While the proportions of LN B cell subsets and CD4 and CD8 T cell subsets were only marginally affected by Sgpl1 deficiency (Fig. S3C), the representation of CD4 effector memory and CD8 central memory cells also increased in LN, albeit to a smaller degree than in spleen (Fig. 4E).

In summary, these data indicate normal thymic T cell development in inducible Sgpl1-deficient mice, but retention of mature T cells in the thymus and LN, leading to reduced T cell numbers in spleen and blood, with concomitant increased proportions of memory T cells.

### Partial Sgpl1-deficiency Promotes CD4^+^Foxp3^+^ T Cells in LN and Spleen

**Figure 5:**
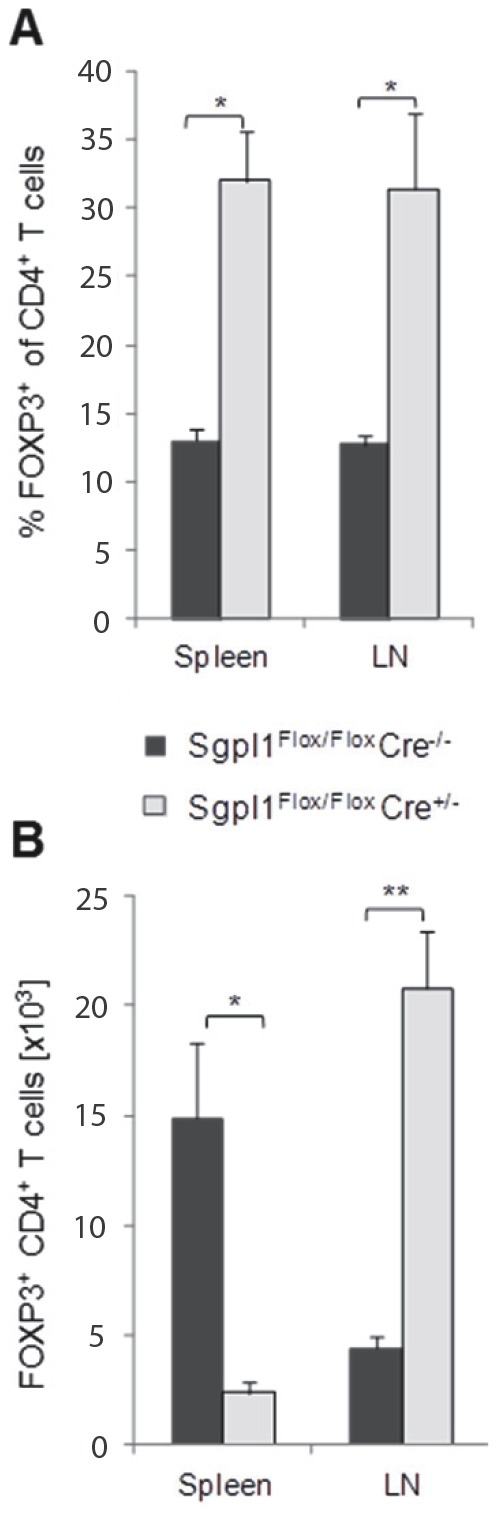
Foxp3^+^ Treg are overrepresented in LN and spleen of in inducible Sgpl1-deficient mice. Two weeks after tamoxifen treatment, cells from blood, LN and spleen were stained for T cell markers and Foxp3. *A,* Mean percentage and *B*, absolute numbers of Foxp3^+^ cells among CD4^+^ T cells (n = 4/group).

CD4 T cells expressing the transcription factor Foxp3 constitute a critically important T regulatory cell subpopulation that dampens inflammatory responses and secures immunological tolerance [29]. Given the modulation of peripheral T cell numbers and composition in inducible Sgpl1-deficient mice, we also assessed the effect on CD4^+^Foxp3^+^ T cells. Partial Sgpl1 deficiency resulted in a profound increase in the proportions of Foxp3^+^ cells in both spleen and LN to more than twice the physiological levels (Fig. 5A). However, due to the marked loss and gain of total overall T cell numbers in spleen and LN, respectively, absolute CD4^+^Foxp3^+^ T cell numbers were 3–4 fold reduced in spleen, whereas they were increased more than 4-fold in LN (Fig. 5B).

### Inducible Sgpl1-deficient Mice are Protected from Delayed-type Hypersensitivity (DTH) Reaction and EAE

**Figure 6:**
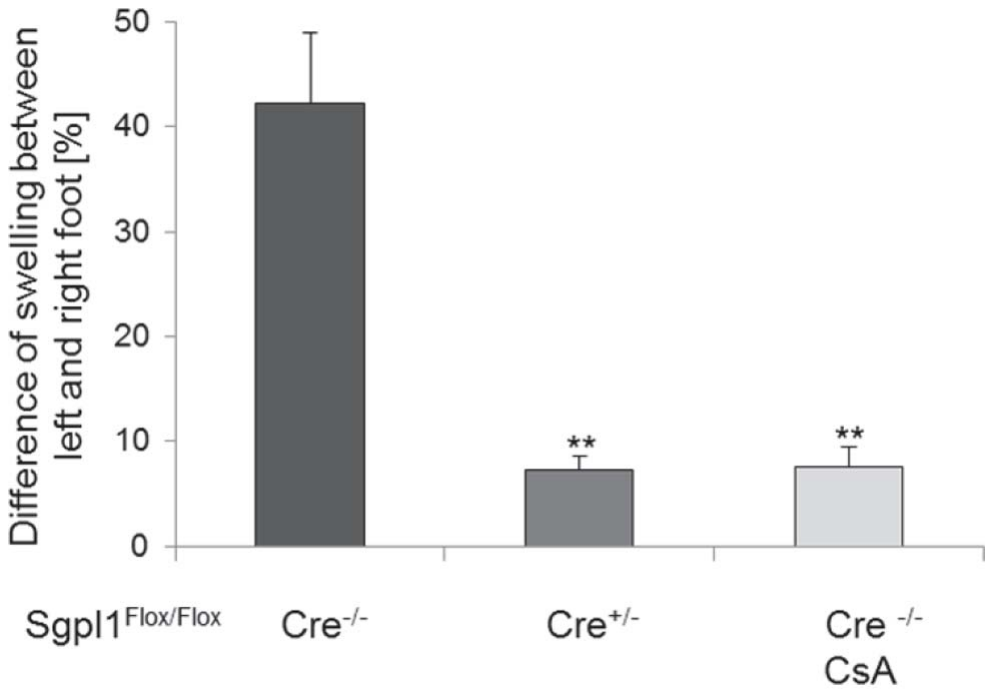
Protection of inducible Sgpl1-deficient mice in DTH. Tamoxifen-induced Sgpl1^Flox/Flox^ Cre^−/−^ mice and Sgpl1^Flox/Flox^ Cre^+/−^ mice (n = 8/group) were immunized with SRBC plus Complete Freund’s Adjuvans s.c. into the left and right flank. Four days after immunization, mice were challenged by s.c. injection of SRBC into the right footpad and PBS was injected into the left footpad. As positive control, Sgpl1^Flox/Flox^ Cre^−/−^ mice (n = 6) received cyclosporin A at the time of challenge. Footpad swelling was measured 24 h after challenge. The difference of swelling of the right *vs.* the left hind paw was measured as specific response to the antigen. Data represent one out of two independent studies with similar outcome.

The capacity of inducible SPL-deficient mice to mount in vivo T cell dependent immune responses was then investigated using the model of DTH induced by systemic immunization and localized challenge with sheep red blood cells (SRBC). Sgpl1^Flox/Flox^ Cre^−/−^ mice developed edema at the site of challenge (footpad), which increased footpad thickness by about 40–50% (Fig. 6; data show one out of two independent studies with similar outcome). Cyclosporin A as a reference compound inhibited the response in these mice by 86%. In induced Sgpl1^Flox/Flox^ Cre^+/−^ mice swelling was reduced as well, with similar inhibition as achieved with Cyclosporin A (87% reduction of swelling *vs.* Sgpl1^Flox/Flox^ Cre^−/−^ mice), indicating pronounced protection by the partial Sgpl1 deficiency. Similar inhibition of DTH has been observed previously also for the S1P1 agonist FTY720 ([30] and our unpublished data).

**Figure 7:**
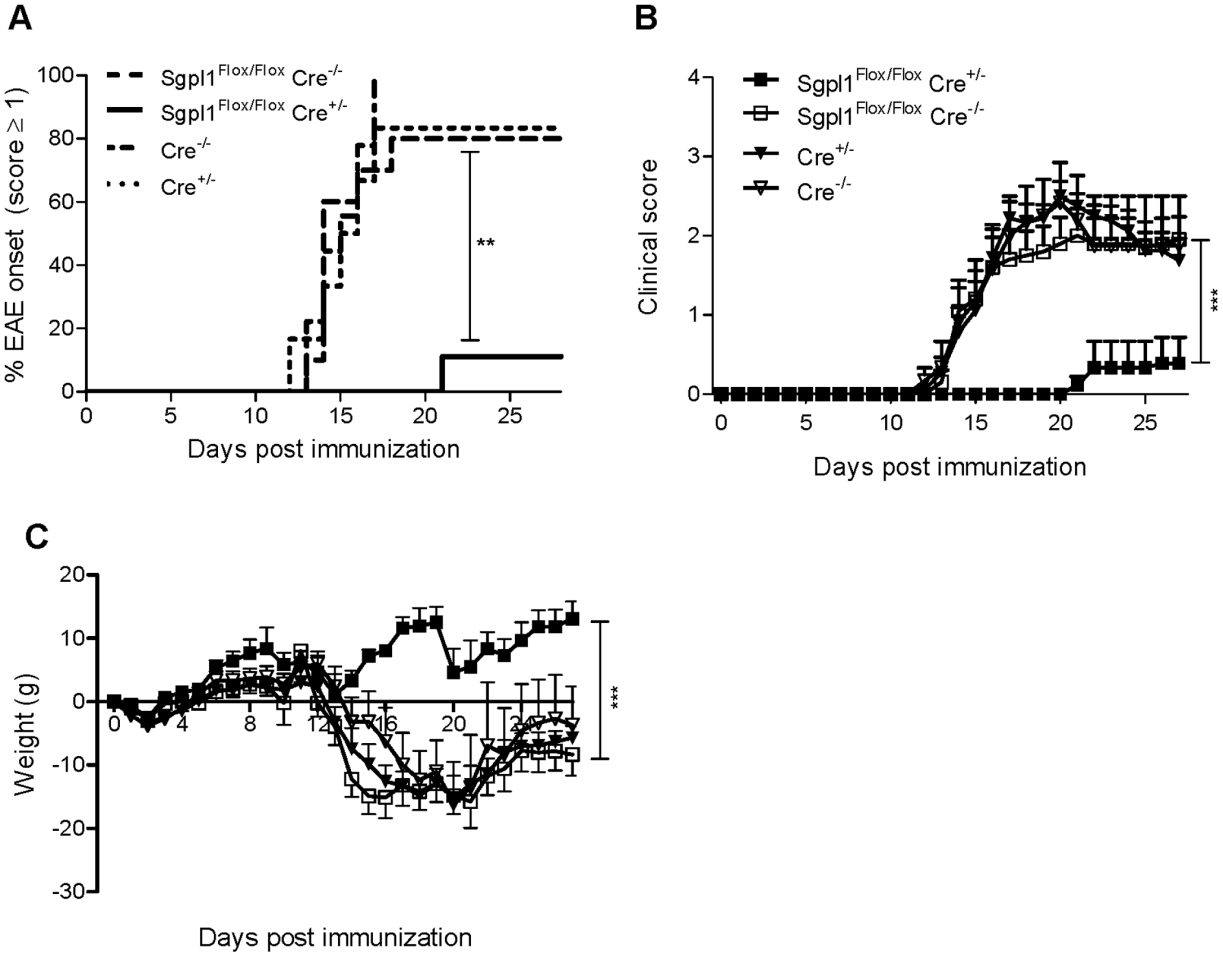
Protection of inducible Sgpl1-deficient mice in EAE. Tamoxifen-induced Sgpl1^Flox/Flox^ Cre^+/−^, Sgpl1^Flox/Flox^ Cre^−/−^, Cre^+/−^, and Cre^−/−^ mice (n = 6–10/group) were immunized with MOG emulsified in Complete Freund’s Adjuvans. Data from one representative experiment out of three independent studies are shown. *A*, Incidence of mice with a clinical EAE score ≥1; *B*, clinical score; *C*, body weight. For histological analysis thoracic sections of spinal cord tissue from Sgpl1^Flox/Flox^ Cre^+/−^ and Sgpl1^Flox/Flox^ Cre^−/−^ mice undergoing EAE (day 24) were stained (*D*) with H&E to visualize CNS-invading cells (scale bar is 500 µm, arrows highlight areas of inflammation); *E*, for CD3^+^ T cells (scale bar is 500 µm, rectangles indicate area of magnification, where scale bar represents 100 µm); and *F*, with solochrome to assess the integrity of the myelin sheath (scale bar is 500 µm, arrows highlight areas of beginning demyeliniation).

**Figure 8:**
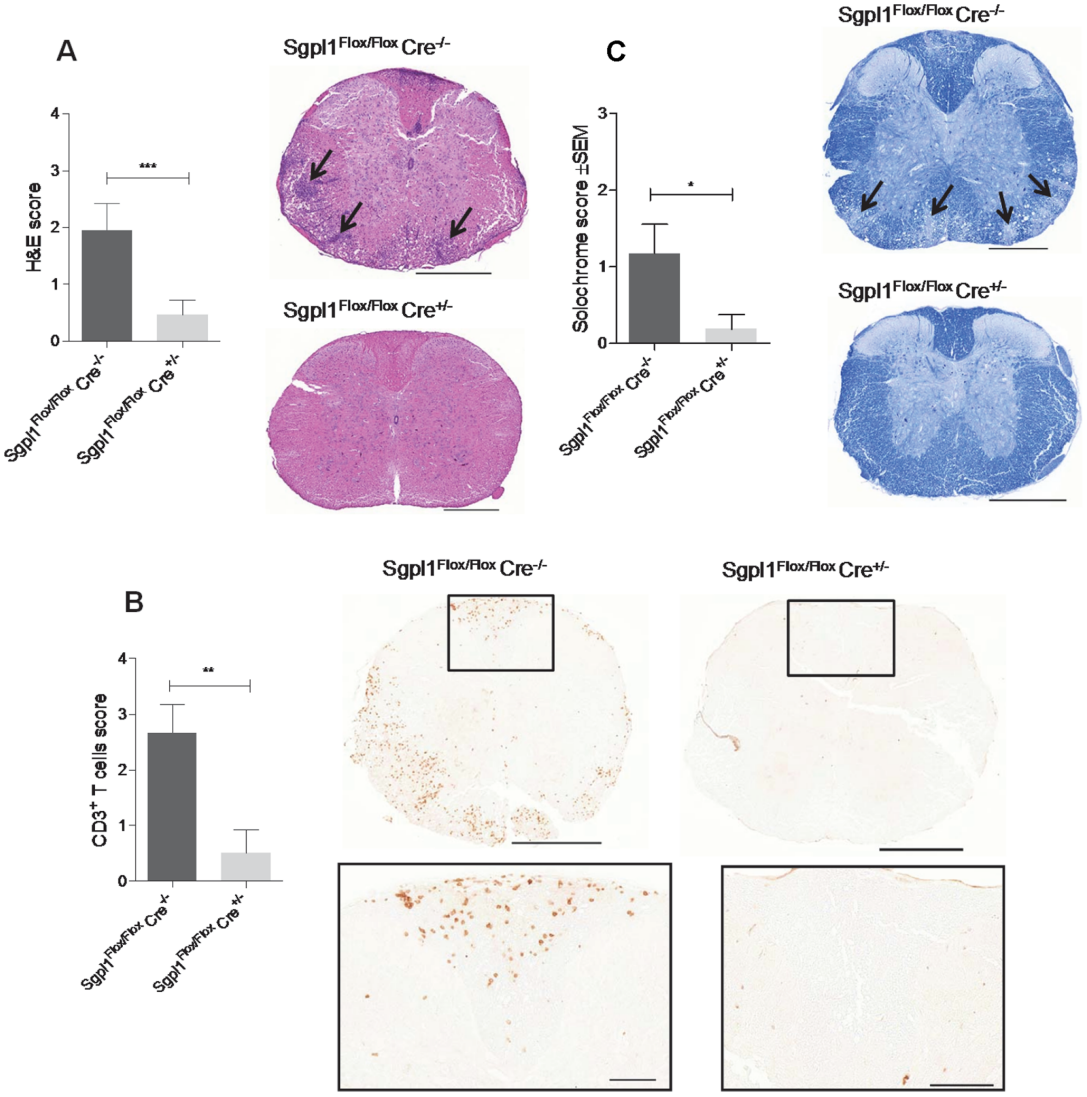
Histology of inducible Sgpl1-deficient mice in EAE. For histological analysis thoracic sections of spinal cord tissue from Sgpl1^Flox/Flox^ Cre^+/−^ and Sgpl1^Flox/Flox^ Cre^−/−^ mice undergoing EAE (day 24) were stained (*A*) with H&E to visualize CNS-invading cells (scale bar is 500 µm, arrows highlight areas of inflammation); *B*, for CD3^+^ T cells (scale bar is 500 µm, rectangles indicate area of magnification, where scale bar represents 100 µm); and *C*, with solochrome to assess the integrity of the myelin sheath (scale bar is 500 µm, arrows highlight areas of beginning demyeliniation).

Since partial Sgpl1 deficiency appears to phenocopy the effect of FTY720 on T cell distribution, we asked if it would also confer protection in murine MOG-induced EAE, a model of multiple sclerosis that depends on the infiltration of pathogenic T cells into the brain. Tamoxifen-treated Sgpl1^Flox/Flox^ Cre^+/−^, Sgpl1^Flox/Flox^ Cre^−/−^, Cre^+/−^, and Cre^−/−^ mice were immunized with MOG in Complete Freund’s Adjuvant and were analyzed daily for clinical signs of EAE over a period of 27 days. Notably, while most animals with normal Sgpl1 expression (Sgpl1^Flox/Flox^ Cre^−/−^, Cre^+/−^, and Cre^−/−^) developed clinical signs of EAE along with a significant loss of body weight (Fig. 7A–C; data from one representative experiment out of three independent studies), Sgpl1-deficient Sgpl1^Flox/Flox^ Cre^+/−^ mice were almost completely protected from EAE. This was accompanied by a markedly reduced histopathological disease score of the spinal cord tissue from Sgpl1^Flox/Flox^ Cre^+/−^ mice compared to control mice, and significantly lower numbers of CNS-invading inflammatory cells, including CD3^+^ T cells (Fig. 8A,B). Furthermore, while a substantial destruction of the myelin sheath was evident on day 24 of EAE in spinal cord tissue of control mice, this was almost undetectable in Sgpl1^Flox/Flox^ Cre^+/−^ mice (Fig. 8C).

**Figure 9:**
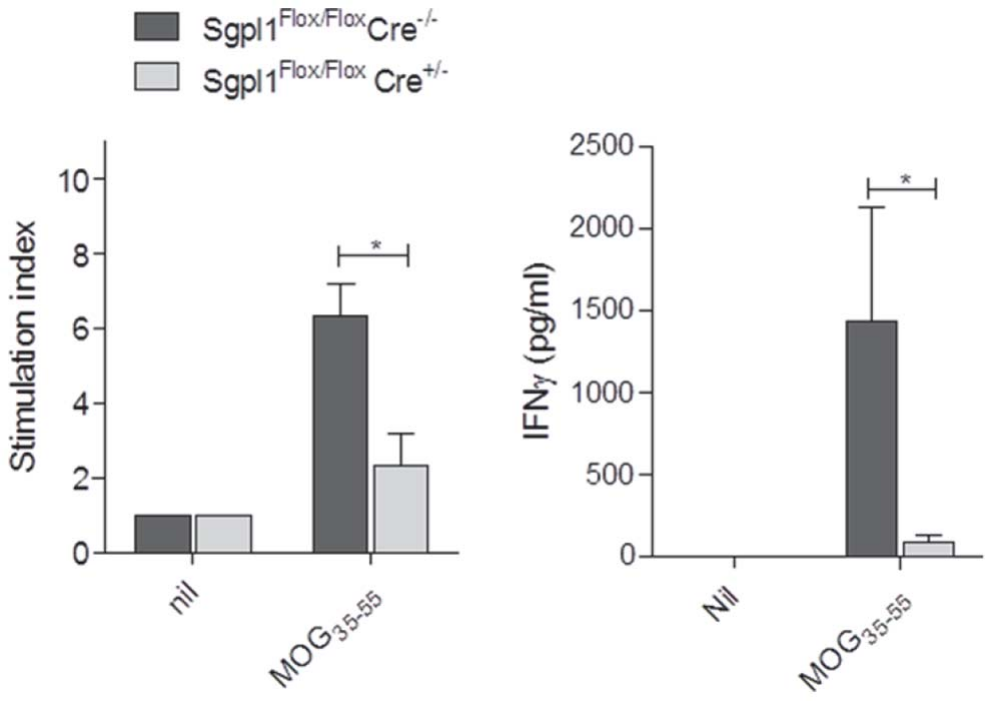
Diminished MOG-specific recall response of T-cells from inducible Sgpl1-deficient mice. CD4-positive T cells purified from Sgpl1^Flox/Flox^ Cre^+/−^ and Sgpl1^Flox/Flox^ Cre^−/−^ mice on day 10 after MOG-induction were restimulated with MOG_35–55_ peptide in the presense of APCs; proliferation and IFNγ secretion was measured. Data from one representative experiment out of three independent studies are shown.

To evaluate whether MOG-immunized Sgpl1^Flox/Flox^ Cre^+/−^ mice were fully capable of mounting a MOG-specific recall response, MOG-dependent T cell proliferation was assessed. First, CD4^+^ T cells were isolated by FACS from MOG-primed Sgpl1^Flox/Flox^ Cre^+/−^ and Sgpl1^Flox/Flox^ Cre^−/−^ mice on day 10 (Fig. S4); then, similar numbers of isolated T cells from both strains were stimulated with MOG_35–55_ peptide in the presence of APCs. T cells isolated from Sgpl1^Flox/Flox^ Cre^+/−^ mice showed significantly reduced proliferation and secreted less IFN-γ than controls (Fig. 9).

## Discussion

In the inducible Sgpl1 KO mice, tamoxifen-induced Cre-mediated gene recombination leads to pronounced, but not complete, downregulation of Sgpl1 activity, typically by 70 to 90% in various tissues. The partial reduction of enzyme activity gives rise to a phenotype that is very different from the short-lived completely Sgpl1-deficient mouse strains [19]–[22]. The inducible knock-out mice develop normally and show no increased mortality over the observation period of 6 months, while featuring reduced numbers of circulating T cells similar to the constitutive KO mice. This is likely due to the fact that the increase of S1P and Sph in the tissues of the inducible KO mice is much less pronounced, namely by factors of about 10 to 300; thus, residual Sgpl1 activity allows turnover of part of the S1P produced in these mice, preventing the vast accumulation of S1P seen in the fully deficient mice and its associated toxicity. Importantly, there is no difference in S1P levels between animals at 2 weeks or 6 months after induction, indicating that the elevated steady-state concentration of S1P in the tissues is maintained permanently. Likewise, the reduction of lymphocytes in the blood persists over this observation period; apparently, the degree of S1P elevation in the secondary lymphoid organs of the inducible Sgpl1-deficient mice is sufficient to prevent lymphocyte egress. These observations are in line with data on humanized Sgpl1 knock-in mice [19] featuring 10–20% residual enzyme activity but still pronounced peripheral reduction of T-lymphocytes counts. Hence, partially Sgpl1-deficient mice may in general be a more suitable genetic model to predict the effect of pharmacological Sgpl1 inhibition which is likely to lead to only partial inhibition of the enzyme as well.

The extent of S1P increase upon partial Sgpl1 deficiency differs considerably between various tissues (Fig. 2B); notably, the highest increase was seen in the spleen and LNs (over 90-fold). This is in line with data from mice treated with the Sgpl1 inhibitors LX2931 [16] and 2-acetyl-4(5)-(1(*R*),2(*S*),3(*R*),4-tetrahydroxybutyl)-imidazole (THI; our unpublished data), which induce the highest S1P increase in the lymphoid organs as well. The extent of S1P increase in different tissues of the inducible Sgpl1-deficient mice does not parallel the extent of reduction of Sgpl1 activity (Fig. 1C) or with baseline Sgpl1 expression in the tissues (data not shown). Therefore, we assume that the importance of Sgpl1 in controlling intracellular S1P levels varies between tissues, e.g., due to different rates of S1P synthesis. Thus, tissues with high S1P production will attain a higher S1P steady level in conditions of partial Sgpl1 deficiency.

In wild-type mice, S1P concentrations are higher in plasma than in the lymphoid organs (Fig. 2A). Importantly, this gradient is inverted in the inducible Sgpl1-deficient mice, i.e. S1P concentrations in the tissues are higher than in the extracellular fluid; thus T cell egress is impaired and their numbers in the circulation are reduced. In the blood of the inducible Sgpl1-deficient mice there is a relatively minor increase in S1P, disproportionate to the one seen in tissues, and no increase in the plasma. This contrasts the situation in fully deficient mice [19], [22] where S1P blood concentrations are highly elevated. By inference, partial Sgpl1 inhibition by a pharmacological inhibitor is not expected to lead to adverse effects via the S1P receptors, e.g., on the heart and on endothelial barriers.

The brain is the only tissue without any increase of S1P, neither in the partial nor the complete Sgpl1-deficient mice. For the inducible Sgpl1-deficient mice this is due to a low recombination frequency in this tissue (Fig. 1A), probably resulting from poor penetration of tamoxifen through the blood-brain barrier [31], and hence no reduction of brain Sgpl1 activity. However, the lack of S1P increase in the brain of constitutive Sgpl1 knock-out mice indicates that Sgpl1 does not control the concentration of S1P in the brain as in other tissues. This finding may be explained as follows: it has been shown that S1P is taken up by cells from erythrocytes via cell-cell contact and that this S1P is then susceptible to cleavage by Sgpl1 [32]; due to the blood-brain barrier, brain tissue is obviously unable to take up S1P from erythrocytes, hence this source of Sgpl1 substrate is missing in the brain making S1P levels unaffected by the absence of Sgpl1. On another note, it has been reported that neurons isolated from constitutive Sgpl1 knock-out mice show elevated S1P, especially after addition of S1P to the cultures which leads to neurotoxicity [33]; in view of our findings, this appears as an experimental setting that obviously does not reflect the *in vivo* situation.

We observed a similar degree of reduction of T-lymphocyte counts in the inducible and constitutive Sgpl1-deficient mice. Using THI as pharmacological inhibitor, it has been shown that the S1P concentration in the spleen required to induce 50% reduction of peripheral lymphocytes is about 4.4 µM [27]; concentrations in the spleen of inducible Sgpl1-deficient mice are considerably higher (about 17 µM), suggesting that an even lower degree of Sgpl1 inhibition may suffice to induce reduction of T cell numbers in the blood. Interestingly, in the inducible deficient mice the effect of Sgpl1 downregulation on blood lymphocyte numbers was confined to the T cells, without any effect on B cells, while in fully Sgpl1-deficient mice B cells in the blood were partially reduced (ref. 19 and data not shown). Although increased B cell numbers in the LN of inducible Sgpl1-deficient mice indicated impaired B cell egress from LN, our data taken together suggest that the migration and distribution of T cells is more strictly controlled by S1P gradients.

As demonstrated before using the S1P modulator drug FTY720, T cells require S1P responsiveness at two major sites; to leave the thymus as mature SP (CD4^+^ or CD8^+^) T cells, and to egress from LN to return to the circulation and home to inflamed tissues [34]. Here we show that partial Sgpl1 deficiency results in over-representation of CD4SP and CD8SP thymocytes, indicating interference with thymic egress comparable to what was reported for FTY720 [35]. Interestingly, this retention occurs without significantly increased ceramide in the thymus which in the case of constitutive KO mice has been proposed to cause abrogation of thymocyte development via apoptosis [36].

The FTY720-induced thymic retention was previously shown to strongly delay the physiological turnover of the peripheral T cell pool and to contribute to an overrepresentation of T cells with a memory phenotype over naïve T cells [37]. Because naïve T cells express the LN homing receptor CD62L and require S1P responsiveness for LN egress, they would be expected to remain more prominently represented in LN than in spleen. This differential effect in LN versus spleen was observed in the present study using partial Sgpl1-deficient mice, similar to previous findings with FTY720-treated mice [38].

In addition to modulating naïve and memory T cell subsets, FTY720 was shown to affect the distribution and accumulation of Foxp3^+^ Tregs in a viral infection model as well as under normal homeostatic conditions in otherwise unchallenged mice (ref. 44 and our unpublished data). We therefore analyzed how the manipulation of the sphingolipid pathway through partial Sgpl1 deficiency affected the distribution of CD4^+^Foxp3^+^ T cells. In both spleen and LN of inducible Sgpl1-deficient mice, CD4 T cell populations were strongly skewed towards Foxp3^+^ cells. This resulted in a weaker decline of total Foxp3^+^ T cells over other T cell subsets in spleen, and it led to a profound gain in absolute Foxp3^+^ T cell numbers in LN. The reasons for these sphingolipid pathway-related specific distribution effects on CD4Foxp3^+^ cells remain to be determined; they might include differential expression of S1P receptors, and/or differences in sensitivity or signaling pathways in response to S1P.

Partial Sgpl1 inhibition was shown here to confer protection in two T cell dependent *in vivo* models, namely in DTH as a classical inflammation model and in EAE as a disease model for multiple sclerosis. In EAE, an almost complete prevention of disease was observed; while spinal cord tissue of control mice undergoing EAE contained significant numbers of CNS-invading CD3^+^ T cells, these cells were undetectable in respective tissue preparations from inducible Sgpl1-deficient mice. The protection observed in these models may be primarily due to the retention of T cells in the LN; however, we cannot exclude that the increased proportion of Foxp3^+^ regulatory CD4^+^ T cells in blood and lymphoid organs and the reduced antigen-responsiveness of the T cells (Fig. 7G) contribute to the protection. These possibilities will need to be addressed in future studies. Interestingly, S1P levels were not elevated in brain and spinal cord of the inducible Sgpl1-deficient mice; hence it appears that the protection in EAE is solely due to the peripheral effect on T cell numbers, quality, and CNS immigration rather than on central effects of S1P on cells in the CNS such as astrocytes that have been described for FTY720 [39].

In conclusion, based on the present data, inhibitors of Sgpl1 may present a new therapeutic option for the treatment of multiple sclerosis. Importantly, the studies on the inducible Sgpl1 KO mice show that partial inhibition of Sgpl1 suffices to induce reduction of peripheral T lymphocyte numbers and to confer protection in T cell dependent models of inflammation, while avoiding the overt toxicity associated with complete KO of the enzyme. Further studies will need to demonstrate whether Sgpl1 inhibitors offer an advantage in terms of efficacy and safety over other agents interfering with S1P-regulated lymphocyte trafficking, in particular S1P_1_ agonists such as FTY720.

## Materials and Methods

### Materials

(E)-(2S,3R)-2-Amino-15-(NBD-amino)-pentadec-4-ene-3-ol-1-phosphate (15-NBD-S1P) was synthesized as described [40]. 1-Hexanoyl-2-[6-NBD-hexanoyl]-*sn*-glycero-3-phosphate (NBD-PA) and standards for LC/MS analytics were obtained from Avanti Polar Lipds (Alabaster, AL). If not indicated otherwise, all other reagents were obtained from Sigma-Aldrich.

### Generation of Sgpl1^−/−^ Mice

Procedures involving animals were conducted in conformity with the guidelines and standards of the Novartis Animal Welfare Organization; studies were approved by the ethics committee of the regional governmental authority “Kantonales Veterinäramt der Stadt Basel” (Permit Numbers: 2119 and 2305). All efforts were made to minimize animal suffering (see in particular details given in section “EAE model”).

To generate homozygous mice with a floxed Sgpl1 gene (Sgpl1^Flox/Flox^), a targeting vector for homologous recombination was designed by cloning 3 kb genomic DNA containing Sgpl1 intron 7, a 250-bp fragment containing Sgpl1 exon 8 as well as 2 kb genomic DNA containing Sgpl1 intron 8, exon 9 and part of intron 9 into vector pRay2loxP2Frt harboring a neomycin expression cassette. Exon 8 was flanked by two loxP elements that facilitate the excision of the exon after breeding with Cre deleter lines. After introduction into C57Bl/6 embryonic stem cells [41], neomycin resistant clones were screened by polymerase chain reaction (PCR) for homologous recombination. Correct targeting was confirmed by Southern blot using a neomycin-specific probe that allowed the exclusion of random integration events of the targeting vector. Selected targeted embryonic stem cells were injected into Balb/c blastocysts and chimeric mice were bred with C57Bl/6 females, resulting in an F1 generation of heterozygous inbred C57Bl/6 mice. To eliminate the FRT-flanked neomycin cassette, Sgpl1 gene targeted mice were crossed with a C57Bl/6 Flp deleter mouse strain and analyzed for the loss of the neomycin cassette. These Sgpl1^Flox/Flox^ mice were further crossed with a C57Bl/6 Cre deleter line [42] to generate the completely Sgpl1-deficient mice.

To generate Tamoxifen-inducible Sgpl1 knock-out mice, the floxed Sgpl1 mice were bred with a B6.C actb-CreERT2 knock-in mouse line (Cre^+/−^); this line had been generated by replacing the floxed EGFP cassette targeted into the β-actin locus by Cre ERT2 [28] and backcrossing to the C57Bl/6 background. Breeding of resulting Sgpl1^Flox/Flox^ Cre^+/−^ with Sgpl1^Flox/Flox^ mice yielded Sgpl1^Flox/Flox^ Cre^+/−^ and Sgpl1^Flox/Flox^ Cre^−/−^ littermates which were used for experimentation. In some experiments, the β-actin CreERT2 mouse line (Cre^+/−^) itself and the corresponding Cre^−/−^ littermates were used as additional control.

All animals used in experiments with inducible knock-out mice were aged 5–7 weeks and were treated with tamoxifen (dissolved in sunflower oil/ethanol (10∶1) mixture at 8 mg/mL) dosed at 40 mg per kg body weight per day, given perorally, once daily on 5 consecutive days.

Analysis of recombination of the Sgpl1 gene and determination of Sgpl1 mRNA expression levels was done as described in Protocol S1.

### Determination of S1P, Sph, and C16-ceramide in Tissues and Blood

Tissue samples were homogenized in water/acetonitrile 1∶1. To 100-µl aliquots of tissue homogenate or body fluid, 25 µl internal standard solution containing 0.4 µg/ml C17-sphingosine, C17-S1P and C17-ceramide were added, followed by 700 µl acetonitrile/methanol/trichloromethane 40∶30∶30. After ultrasonication and a 5 min centrifugation step at 16,200 × g, the upper layer was evaporated to dryness. To reduce unspecific binding the extracts were then subjected to acetylation as described [43].

Analyte concentrations were determined by LC/MS using atmospheric pressure electrospray ionization source on a triple quadrupole mass spectrometer. Instrumentation used was a HTS PAL autoinjector (CTC Analytics, Ziefen, Switzerland), Rheos Allegro (Flux Instruments) and TSQ Quantum Ultra triple quadrupole mass spectrometers, both from Thermo Scientific, Rheinach, Switzerland. For analysis, 10 µl sample was injected on a Reprosil-Pur C18 2.0 × 50 mm reversed-phase column filled with 2.5 µm particles and held at 40°C. For loading, solvent composition was 5% B in A at 100 µl/min for 1 min. For separation a linear, two step gradient from 5% to 60% B in A within 1 min and 60% to 100% B in A within 5 min was applied with a total cycle time of 14 min. During separation the flow rate was held at 200 µl/min. Solvent A was 5 mM ammonium formate and 0.2% formic acid in water and solvent B was 5% methanol in acetonitrile.

Multiple reaction monitoring was used, based on the di- or tri-acetylated precursor ions of the compounds and the corresponding internal standards (Table S1). Sph and ceramide derivatives were detected in positive mode; negative ionization was used for S1P. Quantification was performed based on the area ratios of the compound over internal standard in the extracted ion chromatograms. Recovery was >85% for all analytes. The limit of quantification, as determined by the lowest calibration sample showing signal-to-noise ratio >5 and accuracy <25%, was 10 ng/ml for Sph, 1 ng/ml for S1P, and 25 ng/ml for C16-ceramide.

### Assay of Sgpl1 Activity in Tissues

The method is based on a protocol by Bandhuvula et al. [44] with modifications. Tissues were homogenized in two volumes of lysis buffer (10 mM HEPES, pH 7.4, 100 µM EDTA, 1 mM DTT, 10% (w/v) glycerol, 0.25 M sucrose, protease inhibitor cocktail (Roche)), followed by centrifugation at 500 × g for 5 min. To 5 µL of the lysate 20 µL assay buffer (100 mM HEPES, pH 7.4, 100 µM EDTA, 0.05% Triton X-100, 10 µM pyridoxal-5′-phosphate), and 25 µL 20 µM 15-NBD-S1P were added. The reaction mixture was incubated at 37°C for 30 min, followed by addition of 150 µL 1.33 M KCl in 2.66% HCl, 200 µL methanol containing 0.25 µM NBD-PA as internal standard, and 300 µL chloroform. After mixing and centrifugation, 200 µL of the organic layer was collected and evaporated *in vacuo*. The residue was taken up in 20 µl methanol and a 5 µL-aliquot was injected into the HPLC system (Agilent 1100) equipped with a Luna C18 column (100 × 4.6 mm; Phenomenex). The column was eluted at a flow of 1 ml/min with a gradient of (A) water and (B) methanol/5 mM acetic acid in water/1 M tetrabutylammonium dihydrogenphosphate (Fluka) 95∶4∶1; gradient schedule: 60% B for 1 min; 60 to 100%B for 2.5 min; 100% B for 4.5 min. Fluorescence detection was done at λ_ex_ 485 nm and λ_em_ 530 nm.

### Differential Blood Cell Counts and Flow Cytometry

Differential hematology analysis was performed on whole blood, using an Advia 120 instrument (Siemens, Germany). For flow cytometry analysis of whole blood, erythrocytes were lysed by hypotonic shock, washed once in FACS wash buffer (PBS containing 1% FCS), blocked with mouse Fc Block™ (BD Biosciences), and stained for 30 minutes at 4°C in the dark with the indicated combination of fluorochrome-conjugated mAbs. After staining, the cells were washed twice with wash buffer and resuspended in 200 µl buffer. Samples were analyzed using a FACSCalibur flow cytometer and CellQuest Pro software (BD BioSciences). The following antibodies were used (all from BD BioSciences): anti-CD3-PerCP (clone 145-2C11), anti-CD4-FITC (clone V4), CD8-FITC (clone 53-6.7), and CD19-PerCP (clone 1D3). Absolute cell numbers within each subset were calculated by multiplying their fractional representation determined by FACS by the absolute number of white blood cells measured by hematology analysis. T cells were identified as CD3^+^, and B cells as CD19^+^ mononuclear cells, respectively.

For flow cytometry analysis of thymus, spleen, and lymph nodes (pooled inguinal, axillary and brachial), cell suspensions of each organ were prepared followed by erythrocyte lysis and antibody staining as above. Samples were acquired using a FACSCantoII flow cytometer and analyzed using FlowJo software. Absolute numbers of cells were calculated based on the total cell number in each organ and the proportion of each cell subsets as determined by flow cytometry. Antibody mix for thymic subsets: CD4-PerCP (clone RM4-5, BD) and CD8-PECy7 (clone 53-6.7, ebioscience); germinal center B cells in spleen: PNA-FITC (Vector lab), IgD-PE (clone 11-26c, ebioscience), CD19-PerCP (clone 1D3, BD ), GL7-Alexa647 (clone GL7, ebioscience), B220/CD45-PECy7 (clone RA3-6B2, ebioscience), and CD11b APCeFluor780 (clone M1/70, ebioscience); marginal zone and follicular B cells: IgM-FITC (clone II/41, ebioscence), IgD-PE, CD19-PerCP, CD21/CD35-APC (clone E79, Biolegend), CD23-PECy7 (clone B3B4, Biolegend). Germinal center B cells were identified as CD19^+^B220^+^PNA^+^GL7^+^, marginal zone B cells as CD19^+^CD21/CD35^low^CD23^high^, and follicular B cells as CD19^+^CD21/CD35^high^CD23^dim^.

For memory T cell phenotyping and for detecting Foxp3^+^ cells, cell suspensions the following antibodies wer used: anti-CD3-biotin (clone 145-2C11, BD BioSciences) followed by streptavidin-Alexa Fluor 350 (Invitrogen), anti-CD4-Qdot605 (clone RM4-5, Invitrogen), anti-CD8-APC-H7 (clone 53-6.7, BD Biosciences), anti-CD62L-FITC (clone MEL-14, BD BioSciences), anti-CD44-PE (clone IM7, BD BioSciences), anti-CD25-PECy7 (clone PC61, BD BioSciences), and anti-Foxp3-APC (clone FJK-16s, ebioscience). Following surface staining, cell were permeabilized and stained for the transcription factor Foxp3 using a staining kit (ebioscience). Data were acquired on an LSR II flow cytometer.

### SRBC-induced DTH Reaction

For immunization, Sgpl1^Flox/Flox^ Cre^−^ and Sgpl1^Flox/Flox^ Cre^+^ mice (n = 8/group), used at 8 weeks after tamoxifen treatment, were injected s.c. into the left and right flank with 100 µl, each, of SRBC suspension in PBS (1.7 × 10^9^ cells/mouse), mixed 1∶2 with Complete Freund’s Adjuvant. As positive control, Sgpl1^Flox/Flox^ Cre^−^ mice (n = 6) were treated orally with cyclosporin A (75 mg/kg p.o., dissolved in ethanol and diluted with corn oil 1∶10) at 2 h before and 2 h after the challenge.

Four days after immunization, mice were injected s.c. into the right hind paw with 2×10^8^ SRBC suspended in 50 µl of PBS. The same volume of PBS was injected into the left hind paw. Footpad swelling was measured 24 hours after the challenge using a microscopic lens with a superimposed grid, and was confirmed by weighing of footpads. The degree of DTH reaction was calculated as the percentage of footpad swelling using the following formula: footpad swelling (%) = (thickness of footpad injected with SRBC – thickness of footpad injected with PBS)/thickness of footpad injected with PBC × 100. Data were evaluated using ANOVA, followed by Dunnett’s multiple comparison.

### EAE Model

Sgpl1^Flox/Flox^ Cre^−/−^, Sgpl1^Flox/Flox^ Cre^+/−^, Cre^−/−^, and Cre^+/−^ mice (n = 6–10/group) were used for 6 weeks after tamoxifen treatment. Lightly anesthetized mice were subcutaneously injected with 200 µg MOG protein (Sino Biological) emulsified in incomplete Freund’s Adjuvant supplemented with 4 mg/ml heat-inactivated *Mycobacterium tuberculosis* (both Difco Laboratories). On day 0 and day 2 post immunization, mice were injected i. p. with 200 ng pertussis toxin in PBS.

Animals were scored for neurological signs according the following scale: 0, no symptoms; 1, complete loss of tail tone; 2, clear hind limb weakness; 3, complete hind-limb paralysis; 4, moribund. Animals were sacrificed immediately if they had a clinical score of 4 or after having grade 3.5 (complete bilateral hindlimb paralysis and partial forelimb paralysis) for more than 3 days; also animals having a score of 3 for more than 7 days were sacrificed. If animals needed special care, food and water in form of gel packs was placed in the cages; additionally, the diet was supplemented with a water/nutrient mixture. Animals were sacrificed by inhalation of 5 vol% isoflurane in O_2_ until death occurs, displayed by spontaneous urine loss.

### Histology of Spinal Cords

Deeply anesthetized animals were perfused with PBS followed by 4% paraformaldehyde. Spinal cord tissue was removed and postfixed in 4% paraformaldyde for 24 hours, followed by embedding in paraffin. Representative sections were then taken H&E and solochrome cyanin staining. H&E was used to detect inflammation and to determine the integrity of tissue. Solochrome cyanin staining was used to stain the myelin. Briefly, following dewaxing and rehydration sections for H&E were immersed in Mayer’s hematoxylin for 5 min, followed by rinsing, immersion in eosin for 2 min, rinsed, cleared, and mounted. Sections for solochrome stain were submerged in a solution of 0.2% solochrome cyanine, differentiated in 10% iron alum, followed by rinsing, clearing, and mounting. For immunohistochemistry, tissue was placed in OCT (Tissue-Tek) following PBS perfusion and snap frozen in dry ice cooled isopentane. Frozen sections were fixed in acetone for 10 min at room temperature. Anti-mouse CD3 antibody (Serotec, MCA 1477) was used. Evaluation for extent of H&E-positive inflammatory cells and myelin destruction was done using a semi-quantitative scoring system; 1 = mild; 2 = moderate; 3 = strong. The following CD3 positive staining score was used; 0 = 0–5 cells; 1 = 5–20 cells; score 2 = 20–50 cells; score 3 = 50–100 cells; score 4>100 cells.

### Proliferation Assay and IFNγ Determination

CD3^+^CD4^+^ T cells from spleen and inguinal lymph nodes of MOG-immunized Sgpl1^Flox/Flox^ Cre^−/−^ and Sgpl1^Flox/Flox^ Cre^+/−^ mice (day 10) were isolated by FACS sorting. 40,000 cells were incubated for 72 hrs in RPMI-1640 containing 10% FCS in presence of irradiated APCs (2×10^5^) derived from the same donor mice and MOG_35–55_ (20 µg/ml). 0.5 µCi/well of thymidine was added for the last 24 hrs. Incorporated radioactivity was measured as indicator for proliferation. Supernatants were analyzed for IFN-γ by ELISA (R&D Systems).

### Statistics

Bar graphs in the figures represent average values ± standard error of the mean. Statistical significance was calculated using Student’s T-test, two-tailed for unequal variance, and is indicated in the graphs as follows: *, P<0.05; **, P<0.01; ***, P<0.001; n.s., not significant.

## Supporting Information

Figure S1 **Generation of Sgpl1 KO mice.** *A,* Map of the targeting construct for homologous recombination. *Top:* section of the Sgpl1 locus showing the genomic regions amplified for the generation of the targeting vector. *Bottom:* the Sgpl1 targeting plasmid generated for homologous recombination in ES cells; the Sgpl1 fragment containing exon 8 is flanked by loxP elements. *B,* Schematic representation of the targeted Sgpl1 locus; exon 8 is flanked by loxP elements and can be excised after breeding of targeted mice with a Cre deleter mouse line.
(TIF)

Figure S2 **Sphingolipid concentration in selected tissues of constitutive Sgpl1-deficient mice.** Tissues from constitutive Sgpl1 KO mice (open bars) and from WT littermates (filled bars) (n = 3/group) at two weeks after birth were extracted and sphingolipids were quantified by LC/MS. *A, B*, S1P; *C, D,* Sph; *E*, C16-ceramide. A,C and E show absolute concentrations per weight of tissue. B and D show increase of S1P in the KO mice mice.
(TIF)

Figure S3 **Proportions of cell subsets in lymphoid organs of inducible Sgpl1-deficient or control mice.** *A*, Thymus; *B*, spleen; *C*, LN.
(TIF)

Figure S4 **Purity control of mouse T-cells ny FACS.** CD4-positive T-cells were purified by FACS-sorting from Sgpl1^Flox/Flox^ Cre^+/−^ and Sgpl1^Flox/Flox^ Cre^−/−^ mice on day 10 after MOG-immunization. FACS staining for CD3/CD4 T-cells before and after purification is depicted.
(TIF)

Table S1 **MS parameters of sphingolipid analytes.**
(DOCX)

Protocol S1 (DOCX)
