# Supplementary figures and images for: Partial Deficiency of Sphingosine-1-Phosphate Lyase Confers Protection in Experimental Autoimmune Encephalomyelitis
Source: PLoS One. 2013 Mar 27;8(3):e59630. doi: 10.1371/journal.pone.0059630 (PMC3609791; doi:10.1371/journal.pone.0059630)

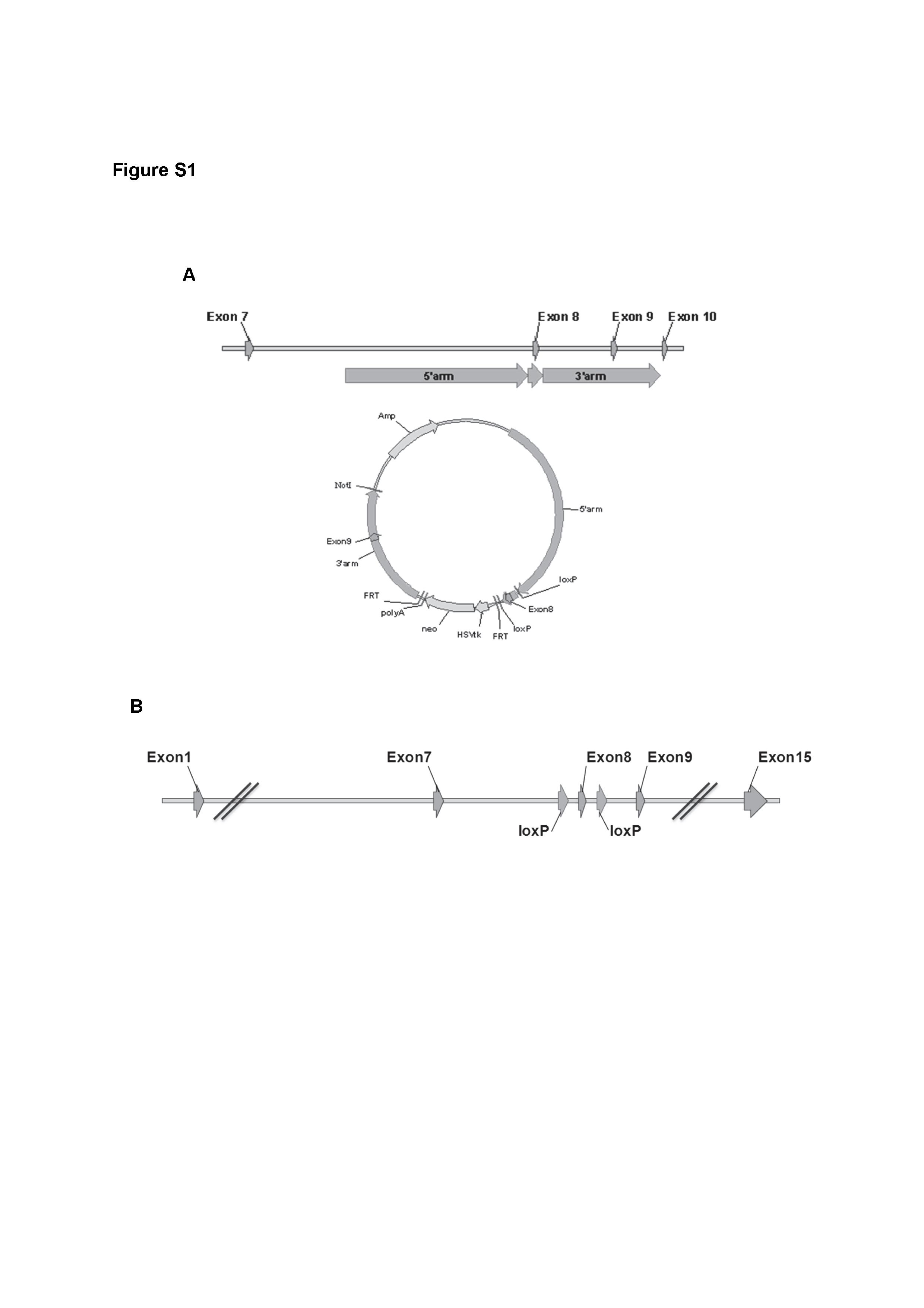

Supplement: Figure S1 — Generation of Sgpl1 KO mice. A, Map of the targeting construct for homologous recombination. Top: section of the Sgpl1 locus showing the genomic regions amplified for the generation of the targeting vector. Bottom: the Sgpl1 targeting plasmid generated for homologous recombination in ES cells; the Sgpl1 fragment containing exon 8 is flanked by loxP elements. B, Schematic representation of the targeted Sgpl1 locus; exon 8 is flanked by loxP elements and can be excised after breeding of targeted mice with a Cre deleter mouse line. (TIF) [file pone.0059630.s001.tif]

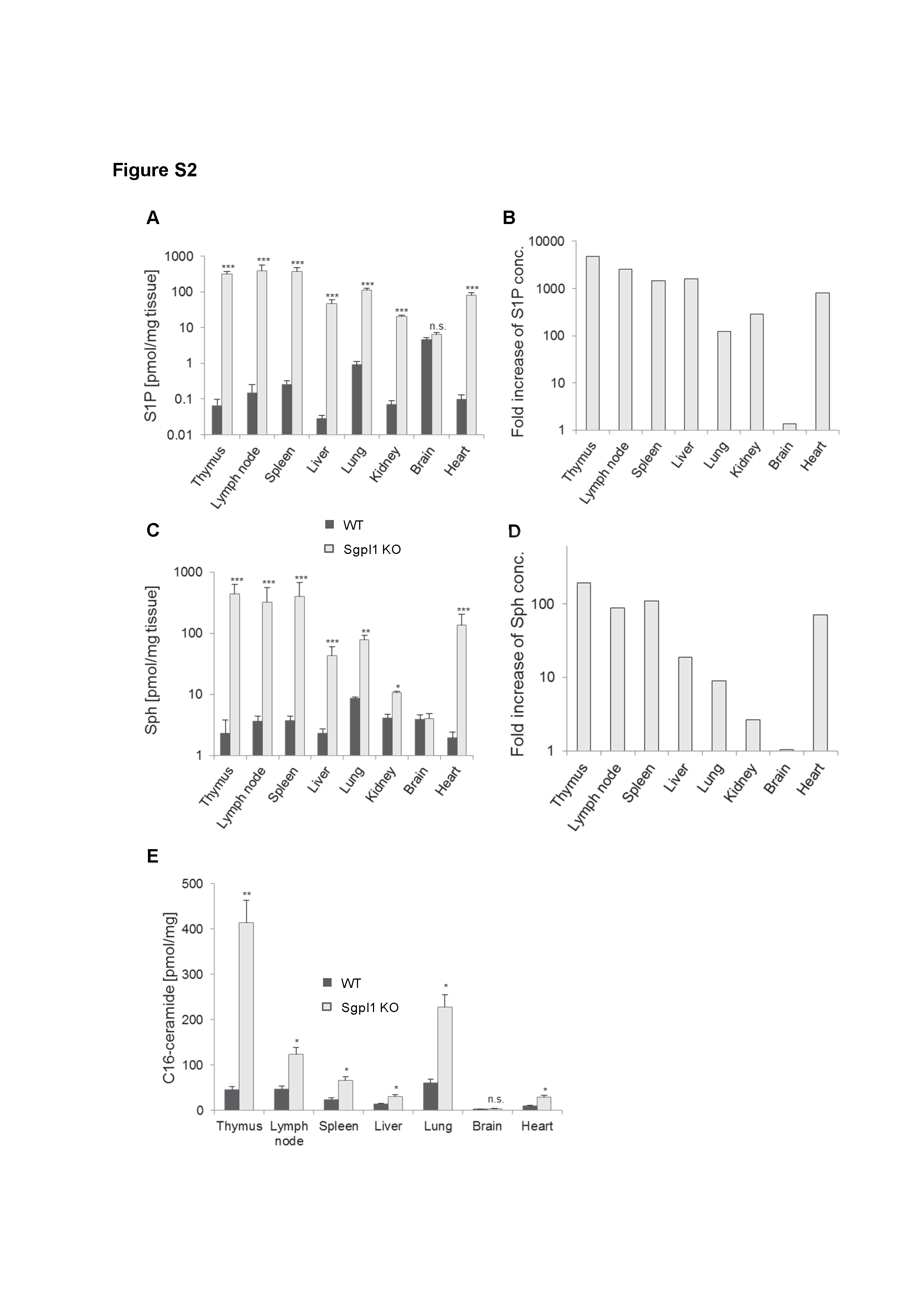

Supplement: Figure S2 — Sphingolipid concentration in selected tissues of constitutive Sgpl1-deficient mice. Tissues from constitutive Sgpl1 KO mice (open bars) and from WT littermates (filled bars) (n = 3/group) at two weeks after birth were extracted and sphingolipids were quantified by LC/MS. A, B, S1P; C, D, Sph; E, C16-ceramide. A,C and E show absolute concentrations per weight of tissue. B and D show increase of S1P in the KO mice mice. (TIF) [file pone.0059630.s002.tif]

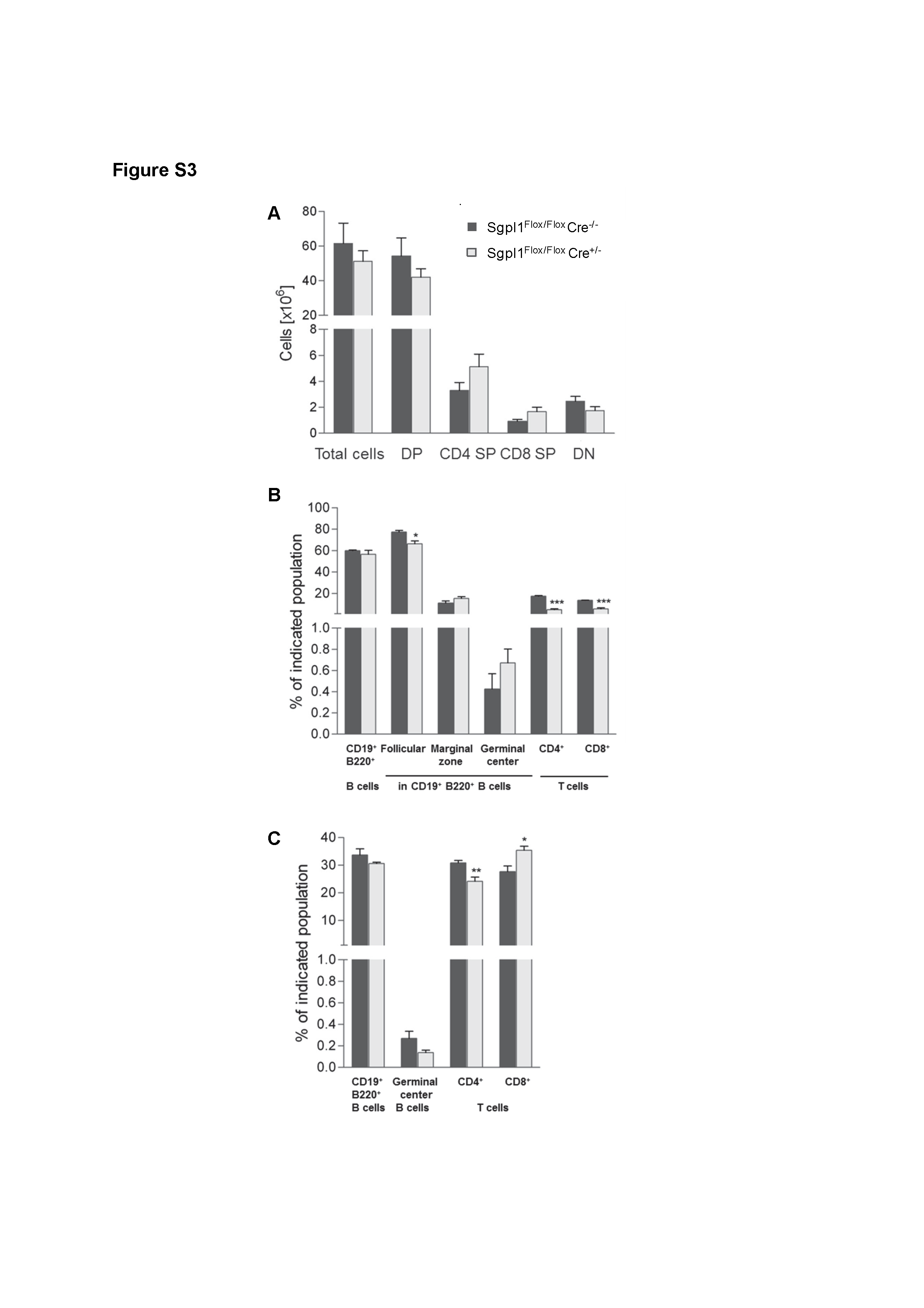

Supplement: Figure S3 — Proportions of cell subsets in lymphoid organs of inducible Sgpl1-deficient or control mice. A, Thymus; B, spleen; C, LN. (TIF) [file pone.0059630.s003.tif]

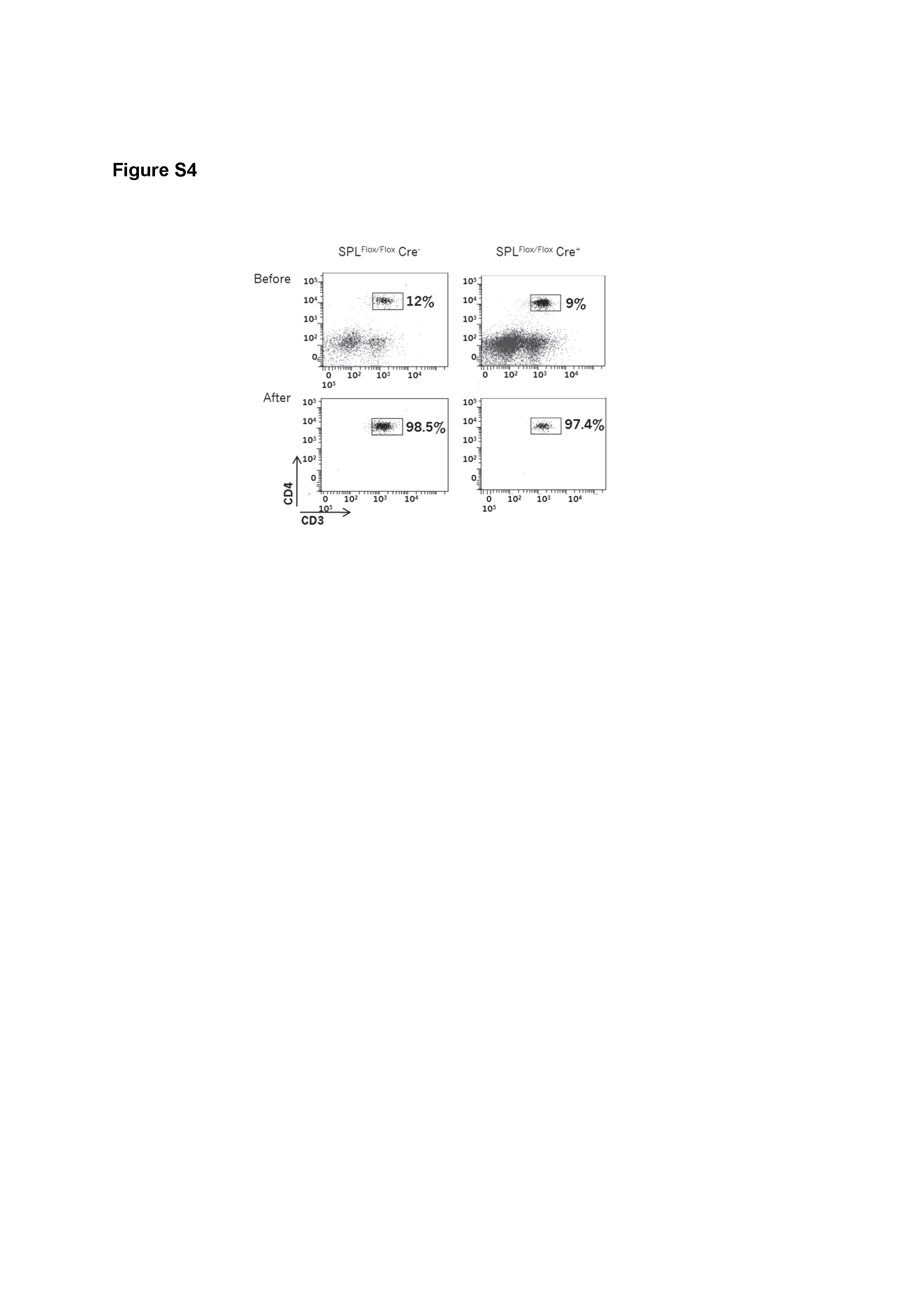

Supplement: Figure S4 — Purity control of mouse T-cells ny FACS. CD4-positive T-cells were purified by FACS-sorting from Sgpl1Flox/Flox Cre+/− and Sgpl1Flox/Flox Cre−/− mice on day 10 after MOG-immunization. FACS staining for CD3/CD4 T-cells before and after purification is depicted. (TIF) [file pone.0059630.s004.tif]
