# Supplementary material for: Partial Deficiency of Sphingosine-1-Phosphate Lyase Confers Protection in Experimental Autoimmune Encephalomyelitis
Source: PLoS One. 2013 Mar 27;8(3):e59630. doi: 10.1371/journal.pone.0059630 (PMC3609791; doi:10.1371/journal.pone.0059630)
Supplement: Table S1 — MS parameters of sphingolipid analytes. (DOCX) [file pone.0059630.s005.docx]

**Table S1 MS parameters of sphingolipid analytes**

| **Analyte** | **Precursor Ion**  **[m/z]** | **Product Ion**  **[m/z]** | **Tube Lens** | **Collision Energy**  **[eV]** |
| --- | --- | --- | --- | --- |
| Sphingosine | 366.2 | 264.2 | 64 | 19 |
| C17-sphingosine * | 352.2 | 250.2 | 66 | 18 |
| Sphingosine-1-phosphate | 504.2 | 402.0 + 462.2 | 92 | 28, 22 |
| C17-sphingosine-1-phosphate * | 490.2 | 388.1 | 94 | 27 |
| C16-ceramide | 562.4 | 264.2 | 97 | 27 |
| C17-ceramide * | 576.4 | 264.2 | 100 | 26 |

* Internal standard
