# Supplementary material for: Partial Deficiency of Sphingosine-1-Phosphate Lyase Confers Protection in Experimental Autoimmune Encephalomyelitis
Source: PLoS One. 2013 Mar 27;8(3):e59630. doi: 10.1371/journal.pone.0059630 (PMC3609791; doi:10.1371/journal.pone.0059630)
Supplement: Protocol S1 — (DOCX) [file pone.0059630.s006.docx]

Analysis of recombination of the Sgpl1 gene

Genomic DNA isolated from mouse tissues served as template in a PCR reaction using primers 5’-CCTGAACCACACTGTCTGTC and 5’-GGAGCAGCAGAGAAGCAGTT. The resulting products of 538 bp (indicating no recombination) and 204 bp (indicating deletion of exon 8) were separated by electrophoresis; gels were stained and band intensity was determined using an AlphaImager HP using Spot Denso software (Alpha Innotech).

Determination of Sgpl1 mRNA expression levels

Taqman RT-PCR was performed using primers 5’-TGATGGCCTGCAAAGCTT and 5’GCCACAATTTCTGGAGTTTTGA, and probe 5’-FAM-CCCTTCTCTAACGCCAAGTCCCGG-BHQ1. Normalization was done to glucocorticoid receptor mRNA amplified using primers 5’-CGGGACCACCTCCCAAA and 5’-CCCCATAATGGCATCCCGAA, and probe 5’-YY-CTTCATCGGAGCACACCAGGCAGA-BHQ1. Data were analyzed using the comparative C_T_ method.
